# Maximizing Interlaminar Fracture Toughness in Bidirectional GFRP through Controlled CNT Heterogeneous Toughening

**DOI:** 10.3390/polym16071011

**Published:** 2024-04-08

**Authors:** Hongchen Zhao, Yunxiao Zhang, Yunfu Ou, Longqiang Wu, Juan Li, Xudan Yao, Xiongwu Yang, Dongsheng Mao

**Affiliations:** 1 Key Laboratory of Marine Materials and Related Technologies, Zhejiang Key Laboratory of Marine Materials and Protective Technologies, Ningbo Institute of Materials Technology and Engineering, Chinese Academy of Sciences, Ningbo 315201, China; zhaohongchen@nimte.ac.cn (H.Z.); zhangyunxiao@nimte.ac.cn (Y.Z.); wulongqiang@nimte.ac.cn (L.W.); yangxiongwu@nimte.ac.cn (X.Y.); 2 School of Materials Science and Chemical Engineering, Ningbo University, Ningbo 315211, China; 3 School of Chemical Sciences, University of Chinese Academy of Sciences, Beijing 100049, China; 4 School of Materials Science and Engineering, NingboTech University, Ningbo 315100, China; lijuan@nbt.edu.cn; 5 School of Aeronautics, Northwestern Polytechnical University, Xi’an 710072, China; x.yao@nwpu.edu.cn

**Keywords:** polymer–matrix composites (PMCs), glass fibers, carbon nanotube, fracture toughness, mechanical testing

## Abstract

“Interleaving” is widely used for interlaminar toughening of fiber-reinforced composites, and the structure of interleaving is one of the important factors affecting the toughening efficiency of laminates. Several experiments have demonstrated that compared to continuous and dense structures, toughening layers with structural heterogeneity can trigger multiple toughening mechanisms and have better toughening effects. On this basis, this work further investigates the application of heterogeneous toughening phases in interlaminar toughening of bidirectional GFRP. CNT was selected to construct toughening phases, which was introduced into the interlaminar of composites through efficient spraying methods. By controlling the amount of CNT, various structures of CNT toughening layers were obtained. The fracture toughness of modified laminates was tested, and their toughening mechanism was analyzed based on fracture surface observation. The results indicate that the optimal CNT usage (0.5 gsm) can increase the initial and extended values of interlayer fracture toughness by 136.0% and 82.0%, respectively. The solvent acetone sprayed with CNT can dissolve and re-precipitate a portion of the sizing agent on the surface of the fibers, which improves the bonding of the fibers to the resin. More importantly, larger discrete particles are formed between the layers, guiding the cracks to deflect in the orientation of the toughened layer. This generates additional energy dissipation and ultimately presents an optimal toughening effect.

## 1. Introduction

In recent years, great efforts have been made in improving the interlaminar fracture toughness (IFT) of glass fiber-reinforced plastic (GFRP). Among so many methods, interleaving has widely attracted attention for its effectiveness and convenience [1,2]. This strategy was proposed in the 20th century; it separates interlaminar regions from the overall composite and provides controllable and targeted toughening [3,4,5]. It can achieve significant toughening and impact resistance while maintaining easy and convenient processing [6]. One of the key factors for interlaminar toughening is the selection and design of toughening layers [7,8,9]. Carbon nanotubes (CNTs) are considered as ideal fillers for enhancing, toughening, and functionalizing composites, due to their large aspect ratio, high specific surface area, good chemical stability, and excellent mechanical and electrical properties [10,11,12,13,14].

The mode Ⅰ toughening efficiency of laminates is highly sensitive to the structure characteristic of CNT interleaves [15,16], such as thickness [17], morphology [18,19], orientation [17,20], and distribution [14,19]. In previous research, various forms of CNTs like carbon nanotube veils [21], carbon nanotube films [22], and buckpaper [23,24] have been used as toughening layers. However, there are few reports of significant improvement of mode I IFT based on CNT toughening. Dai [21], Liu et al. [25] introduced CNT films and CO/CNT composite films (thickness: 30–40 µm) between composite layers, but this led to a decrease in mode I interlaminar fracture toughness (G_IC_). Ni [26] et al. further improved the interlaminar distribution of CNTs. They used long CNTs to create a “nano-stitched” structure in which the CNTs were orientated perpendicular to the laminate. However, the G_ⅠC_ of the toughened samples showed no difference from the baseline.

Plenty of evidence indicates that compared to continuous and dense structures, heterogeneous toughening layers can trigger multiple toughening mechanisms and have better toughening effects [27,28,29,30]. Zhang et al. [31] designed a discrete thermoplastic microphase in the interlaminar of composite, which resulted in about a 49% increase in mode I interlaminar fracture toughness propagation value (G_IC,prop_) at a trace addition of 0.015 wt%. In this composite system, it has been proven that the dispersed PMMA points can guide crack growth. In Abidin’s research [32], it was found that CNTs uniformly distributed in the matrix cannot fully utilize their toughening potential. Heterogeneous composites with an average distance of approximately 40 μm between CNT-enriched regions are recommended.

A simple and accessible way to modulate the structure of interleaves of composite is to vary the amount of CNT. In previous studies [15], we found that it is feasible to deposit CNT particles of controlled size on unidirectional carbon fiber cloth by spraying. Lower contents of CNTs were able to aggregate into discrete clusters, while higher-surface-density CNT particles would stack into a continuous film. Ultimately, the results were as expected; the dispersed CNT particles showed the optimal toughening effect in the interlaminar. However, for woven GFRP, the toughening mechanism of the laminates will change due to the influence of the bidirectional woven structure. Therefore, the usability of “CNT particle toughening” to this system remains to be investigated.

This work was proposed to address this issue, exploring the effect of CNT interleaf structure on the interlaminar toughening efficiency in GFRP systems. Carbon nanotube (length: 0.5–2 μm) additions of 0–1.0 gsm were selected to be uniformly dispersed in acetone by microjet to obtain individual particles. The dispersion was further sprayed on glass fiber fabrics, where some of the particles would re-aggregate with the evaporation of the solvent. Thereby, CNT toughening layers with various aggregation structures like discrete aggregated particles or continuous films are constructed on the surface of glass fiber. The corresponding GFRP-toughened samples were eventually prepared. Their interlaminar toughening efficiency was tested using the Double Cantilever Beam (DCB) and the toughening mechanisms were comparatively analyzed using advanced surface analysis methods.

## 2. Materials and Methods

### 2.1. Materials

The non-alkali glass fiber plain weave fabric (EWR-400) used in this study had a surface density of 400 g/m^2^ and was obtained from China Giant Glass Fiber Co., Ltd. (Tongxiang, China). The epoxy resin, EPONTM Resin 862 (diglycidyl ether of bisphenol F), was purchased from Hexion Inc. (Columbus, OH, USA), characterized by low viscosity and good wetting properties. The curing agent, Polyetheramine D-230, was acquired from Aladdin (Shanghai, China) Chemical Co., Ltd. High-purity (>98 wt%) multi-walled CNT powders without surface modification (TNSM1) were obtained from Chengdu Times Nano Co., Ltd. (Chengdu, China), with length ranging from 0.5 to 2 μm and diameter ranging from 5 to 15 nm.

### 2.2. Surface Modification

**Figure 1:**
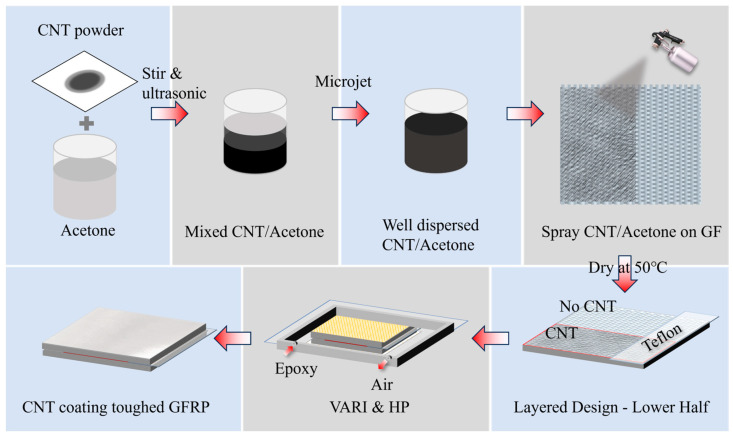
Flow chart of glass fiber modification via CNT and the process of laminate preparation.

The process depicted in Figure 1 illustrates how to effectively disperse CNT powder in acetone and spray on glass fiber woven fabric. Firstly, 0.5 g of CNT powder was mixed with 200 g acetone and simply stirred. Next, the mixture was treated with an ultrasonic device (cell crusher, JY92 series, equipped with 10 mm diameter flat head longitudinal vibration tips, Shanghai Huxi Industrial Co., Ltd. Shanghai, China) for 20 min at a power of 500 W, then stirred evenly, treated ultrasonically again, and stirred twice more to realize coarse dispersion. After that, we applied microjet processing for at least 5 rounds to achieve a fine dispersion, in which the CNT/acetone solution was squeezed through a 100 μm diameter pore at a pressure of 150 MPa, generating a fluid several times the speed of sound with huge shear force and achieving homogeneous dispersion. The way to determine good dispersion is to stabilize the dispersion pressure within the cycle at 150 MPa, and the CNT/acetone solution will not stratify within 30 min. The resulting mixture of acetone and CNT was than evenly sprayed onto the surfaces of GF woven plain fabric with an airbrush. The air pressure was set at 0.75 MPa, and the spraying nozzle was positioned 30 cm away from the fabric. Finally, the CNT-modified fabrics were dried in a vacuum oven at 50 °C for 12 h to remove all residual acetone. The content of acetone dispersion is controlled to obtain CNT modification layers with different area densities on glass fabrics: 0.3, 0.5, 0.7, and 1 gsm. In addition, a baseline without spray treatment and samples only treated with 200 gsm acetone were set up (named 0 gsm).

### 2.3. Laminate Preparation

A laboratory-optimized vacuum-assisted resin infusion (VARI) process and hot press curing process were adopted to make the CNT spray coating for toughened GFRP. Specifically, the glass fabric was cut into a size of 300 mm × 300 mm and arranged to build a preform of [0°]_26_, and the 15th and 16th layers were replaced with CNT spray coating-modified layers. With the help of a vacuum and two layers of infusion media added on top and bottom, the infusion process can realize a good resin impregnation. When finishing the infusion process, the preform was then transferred to a flat vulcanizing machine and cured at 80 °C/1 MPa for 2 h and 120 °C /1 MPa for another 2 h, with a warming process of 20 min between 80 and 120 °C. After cooling down to room temperature, the resulting laminates with a thickness of about 5.8 mm were cut into 6 different groups of samples, divided by the contain of CNT.

### 2.4. Fracture Tests

**Figure 2:**
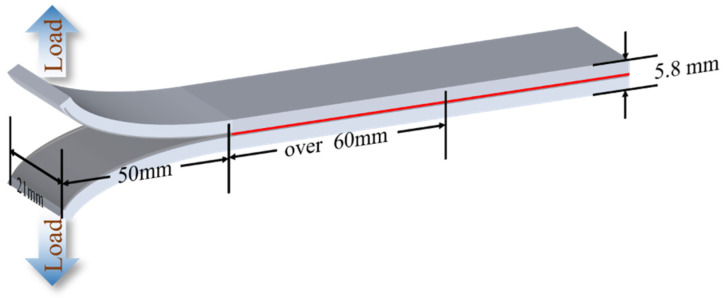
DCB sample in mode Ⅰ interlaminar fracture toughness test.

The interlaminar fracture properties of laminates were evaluated through the DCB tests according to ASTM Standards D5528-01 with a sample size of 250 mm × 21 mm × 6.6 mm (Figure 2). A universal testing machine (Z1.0, ZwickRoell GmbH & Co.KG, Ulm, Germany) with a 1 kN load cell at a crosshead speed of 1 mm/min was employed in the test, and the Modified Beam Theory (MBT) method was chosen to calculate the mode Ⅰ strain energy release rate (G_I_) through the following equation:

$$G_{I}=\frac{3\;\times \;F\;\times \;\delta }{2\;\times \;w\;\times \;(a+\left|\Delta \right|)}$$

where F is the load, δ represents the opening displacement at the loading point, w stands for the sample width, a is the crack extension distance, and Δ is the intercept of the fitted line a-C^1/3^. Here, C represents the ratio of the opening displacement to the load δ/F. To ensure data stability, a minimum of 5 samples of each mode were tested.

### 2.5. Other Characterizations

A scanning electron microscope (SEM, Hitachi Regulus 8230, Tokyo, Japan) was utilized to observe the interlaminar fracture interface and silicon wafer, which was sprayed with a very small amount of CNTs that dispersed well in acetone. SEM acceleration voltage was set at 4.0 kV, and the surfaces were platinum-coated (180 s, 9 nm thickness).

A small amount of dispersed CNT/acetone solution was dropped onto the carbon support membrane. After the acetone was totally evaporated, the microstructure of carbon nanotubes was studied using high-resolution transmission electron microscopy (HRTEM, Talos F200x, Thermo Fisher, Waltham, MA, USA). The accelerating voltage of the HRTEM was set at 200 kV.

Raman spectroscopy (Renishaw, London, UK) with 532 nm laser and 1800 L/mm grating was used to analyze the type and quality of CNTs.

The morphology of glass fiber surface and carbon nanotube spray layer was observed by scanning probe microscopy (SPM, Bruker Dimension ICON, Billerica, MA, USA), using atomic force microscopy method and tapping mode. The interaction force between the probe and sample surface was detected, with a driving amplitude of 700 mV and an amplitude set point of 350 mV. We also used SPM images to calculate fiber surface roughness, selecting an area of 3 × 3 microns in the center of the AFM image and reading the Ra results from the matching software testing.

## 3. Result and Discussion

### 3.1. Characterization of Short CNT

**Figure 3:**
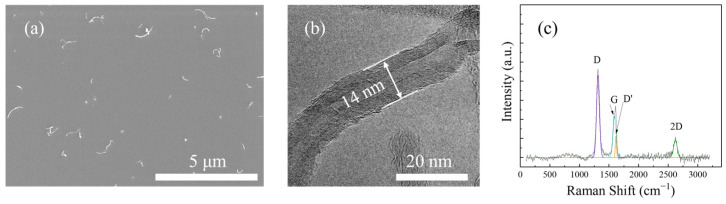
(**a**) SEM and (**b**) TEM images of short CNT; (**c**) a typical Raman spectrum of short CNT powders.

In previous work [15], it was found that spraying short CNTs of 0.5–2 µm on fiber fabrics can give controlled aggregated structures, while long CNTs are prone to film formation. Therefore, in this article, short CNTs were chosen as toughening fillers. Figure 3a,b show the morphology of CNTs, which are 14 nm wide soft filaments with an average length of no more than 2 µm. TEM and Raman spectroscopy revealed the multi-walled structure inside CNTs.

### 3.2. Surface Morphology of CNT-Coated Glass Fiber Fabrics

**Figure 4:**
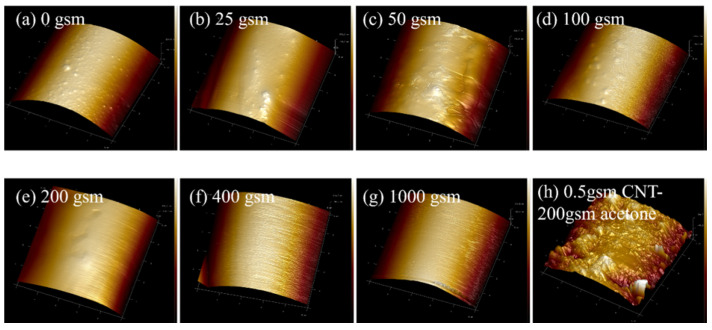
GF surface treated with different amounts of acetone spray and CNT/acetone dispersion spray.

**Figure 5:**
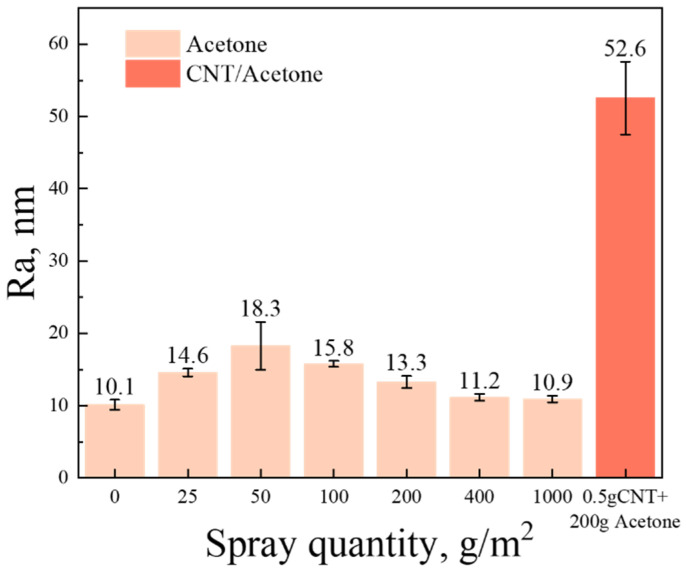
Statistical results of GF surface roughness.

**Figure 6:**
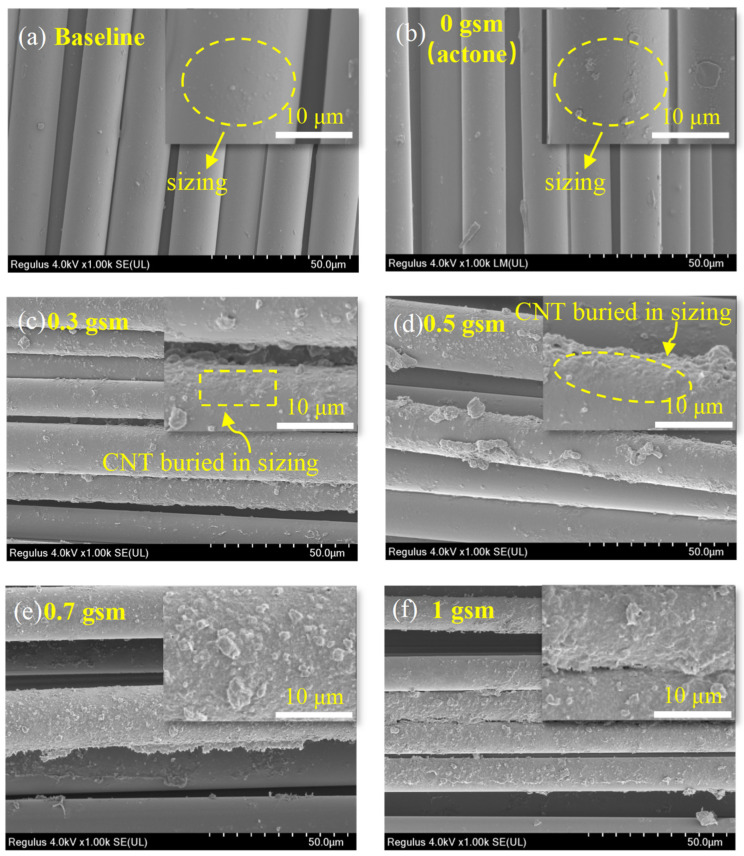
SEM images of glass fabrics and CNT spray coating: (**a**) is commercial glass fabrics without any modification; (**b**–**f**) are fabrics coated with short CNT with areal densities of 0, 0.3, 0.5, 0.7, and 1.0 gsm.

Acetone or acetone-dispersed CNTs were applied to the surface of glass fabrics by spraying method, and the morphology of the samples is shown in Figure 4. The surface of the baseline is relatively smooth but not clean, with a small number of bubbly protrusions (Figure 4a). This is attributed to the coverage of the sizing agent. Interestingly, it was found that acetone can dissolve the sizing agent on the surface of glass fibers through SPM observation. And the degree of dissolution is related to the quantity of acetone, which further affects the morphology of the fiber surface. (To ensure the stability of the spraying process, the concentration of CNT dispersion was controlled to a certain extent in this experiment). Therefore, for 0 gsm samples, the sizing agent on the fibers partially dissolves after being treated by 200 gsm acetone (Figure 4e). This results in more uneven protrusions, leading to a rougher surface (Figure 5 and Figure 6b).

Spraying CNT dispersion onto the surface of glass fiber significantly improves its surface roughness (Figure 4h and Figure 5). Along with the volatilization of acetone, CNTs tend to aggregate with each other. When the content of CNTs is low, some of them aggregate to form discrete agglomerated particles, as shown in Figure 6c,d. And compared to 0.3 gsm, the area density of 0.5 gsm corresponds to larger CNT aggregation particles (Figure 6c,d). However, with the introduction of more CNTs, continuous CNT membranes are formed, and the glass fibers are completely covered (Figure 6e,f). In addition, it is observed that in local areas, the CNTs on the surface of fibers are coated with sizing agents. When spraying CNT dispersion onto glass fabrics, the acetone contained in it would also be sprayed on the fiber surface and dissolve some of the sizing agent. As the solvent evaporates, some sizing agents re-precipitate and adhere to the fiber surface or around the CNT. This is beneficial for the good bonding between glass fibers and resin.

### 3.3. Mode I Interlaminar Fracture Toughness

**Figure 7:**
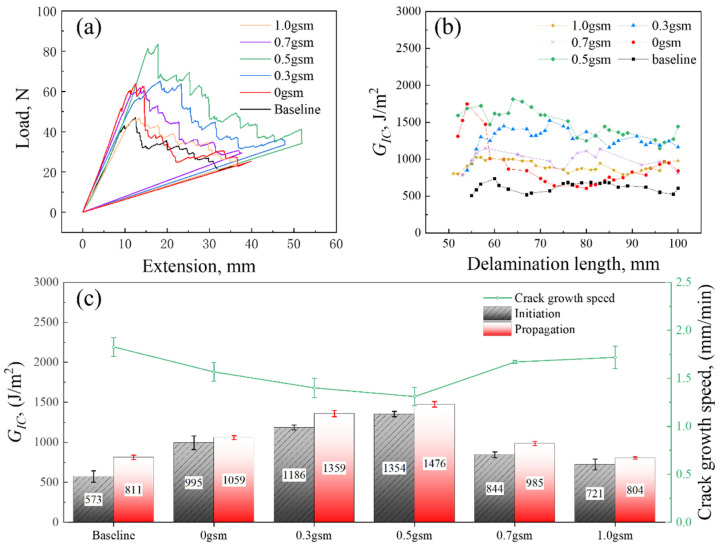
Representative load-opening displacement curves (**a**) and R-curves (**b**) of specimens with and without CNT spray coating; (**c**) is the comparison of crack growth speed, initiation, and propagation value of G_IC_.

The samples were subjected to DCB testing to obtain their mode I interlaminar fracture toughness results (G_IC_), as shown in Figure 7. And the G_IC_ was found to be improved in all spray-treated laminates. Interestingly, the samples treated with acetone only also exhibit an increase of 73.6% and 30.6% in G_IC,ini_ and G_IC,prop_. Furthermore, the introduction of a small amount of CNTs can significantly improve the fracture toughness of the GFRP samples, wherein the CNT-toughened layer with area density of 0.3 gsm shows 107.0% and 67.6% enhancement in G_IC,ini_ and G_IC,prop_, while the 0.5 gsm CNT-toughened layer shows the best toughening effect, with about 136.0% and 82.0% enhancement in G_IC,ini_ and G_IC,prop_. However, as the CNT in interlaminar continues to accumulate, its toughening efficiency is rather restrained. The increase in G_IC,prop_ values of the 0.7 gsm samples is less than 21.5%, and 1 gsm samples are even lower than that of the 0 gsm samples.

It was observed that the R curves of all samples showed an overall flat or decreasing trend, indicating the absence of fiber bridging (Figure 7b), which is different from the previous experiments of CFRP [15]. In addition, it is noteworthy that the 0, 0.3, and 0.5 gsm samples exhibit relatively high initial values of fracture toughness. However, as shown in the load–displacement curve and the R curve, there is a stepwise decrease in 0 gsm, with a higher crack extension rate, ultimately leading to a lower propagation value of fracture toughness (Figure 7c). This may be attributed to the rapid development after encountering and breaking through obstacles during crack propagation. The R curves of the 0.3 and 0.5 gsm samples showed significant local fluctuations but remained flat overall, revealing a more stable and complex process of delamination failure, resulting in higher propagation fracture toughness.

### 3.4. Fractography and Toughening Mechanisms

**Figure 8:**
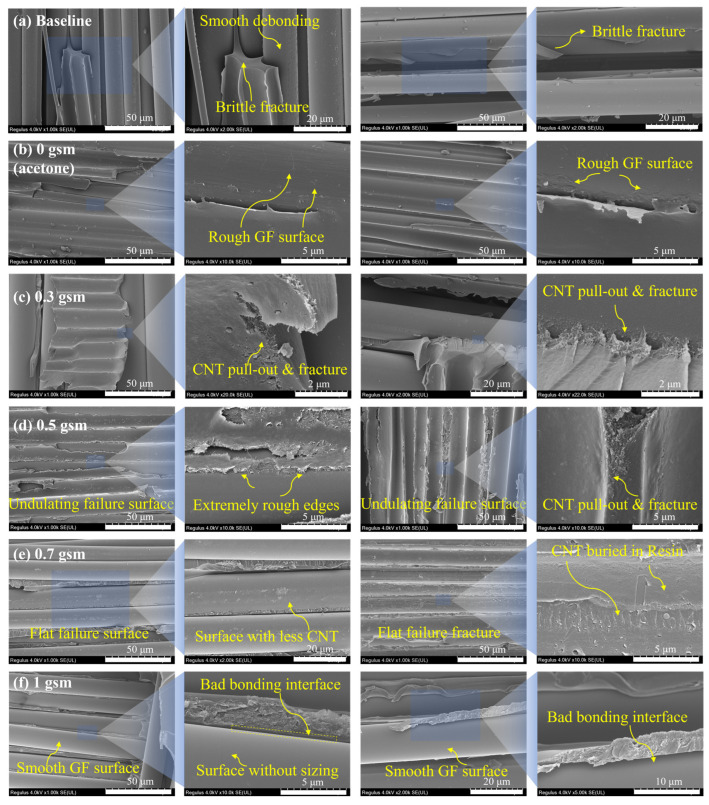
Comparison of fracture surfaces: (**a**) baseline sample without CNT modification; (**b**–**f**) samples modified with 0, 0.3, 0.5, 0.7, and 1.0 gsm CNT spray coating.

**Figure 9:**
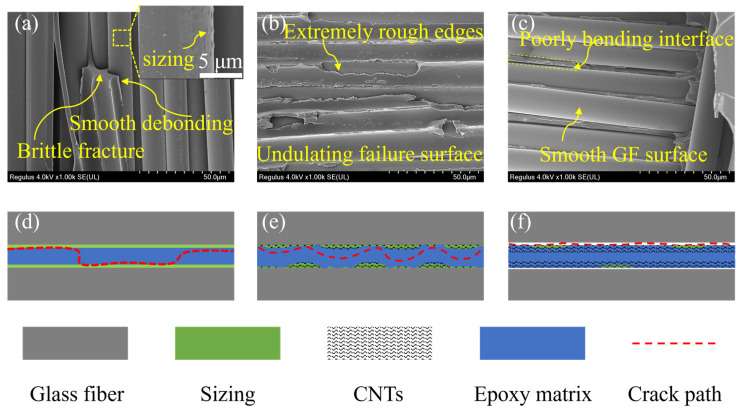
Possible modes of crack propagation in laminates with control sample (**a**,**d**) and CNT-toughened samples whose areal densities are (**b**,**e**) 0.5 and (**c**,**f**) 1.0 gsm, respectively.

Observing the fracture surface of samples after the DCB test, their fracture mechanisms were preliminarily analyzed. As shown in Figure 8, the resin of the baseline exhibits typical brittle fracture. A large quantity of exposed fibers are present, whose surface is smooth and tidy, revealing poor bonding between them and the resin matrix (Figure 8a). Therefore, the main form of failure was debonding at the fiber–resin interface; the schematic diagram is shown in Figure 9d.

After the acetone spray treatment, better bonding was observed at the interface between fiber and resin (Figure 8b), which was attributed to a slight increase in surface roughness of fibers due to partial dissolution of the sizing agent, as mentioned in Section 3.2. In the samples modified with CNT, pull-out, fracture, and bridging were observed, which led to additional energy dissipation (Figure 8c–f). Moreover, in the vicinity of the CNT nanoparticles, a messy and rough morphology is presented, which suggests that at the nanoscale, the CNT can induce the propagation path of surrounding cracks, making it more complex. Overall, an intrinsic toughening effect was achieved.

Improved bonding between fibers and matrix is observed in the fracture surface of 0.3 and 0.5 gsm samples (Figure 8c,d), which is attributed to the bonding of the re-precipitated sizing agent and considerable increase in fiber surface roughness. This leads to more energy dissipation as the cracks expand along the fiber–resin interface, and fiber debonding is suppressed. In addition, a particularly complex and undulating failure surface was observed in the 0.5 gsm samples (Figure 8d). The resin is torn discontinuously, with extremely rough edges, and covered with tangled and intertwined CNTs. This is due to the clustering of CNTs into larger micro-sized particles that are discretely distributed between layers at a surface density of 0.5 gsm. These dispersed CNT particles can guide cracks to undergo multiple orientation deflections at the micrometer scale in interlaminar, leading to an increased crack propagation path. Therefore, its delamination failure mainly occurs in the toughened layer, and it has complex crack propagation under the influence of CNT (Figure 9e).

By contrast, the SEM images of the 0.7 and 1 gsm samples showed smoother fiber debonding surfaces (Figure 8e,f). Especially for the 1 gsm sample, the fiber surface is even cleaner compared to baseline (Figure 8a,f). This is because, with more CNT spraying, a large amount of acetone is also sprayed onto the surface of the glass fiber, and the sizing agent is almost completely dissolved and washed. As revealed in Figure 4 above, the fiber surface becomes particularly clean and smooth, which has an adverse impact on the bonding of glass fibers with CNT and resin. And in these samples, too many CNTs are entangled with each other, aggregating into continuous films in the interlaminar. Cracks do not readily extend within them, and the interlaminar crossing of cracks is even suppressed. Therefore, cracks are prone to deflection and remain at weaker fiber–resin interfaces, ultimately leading to severe fiber debonding (Figure 9f).

From the above, it is found that there are two keys to efficient toughening using CNT interleaves: the well-bonded fiber–resin interface and the complication of crack propagation paths in the interlaminar region.

In terms of constructing a good interface, the reasonable coordination between acetone and sizing agent is extremely important. When modifying glass fibers with a lower content of CNT dispersion (0–0.5 gsm), an appropriate amount of acetone is sprayed on its surface. Thereby, CNT can be adhered to the fiber surface through the re-precipitation of the sizing agent. This is good for further improving the surface roughness of fibers and the tight and effective adhesion of CNTs on fibers, achieving an improved fiber–resin interface. When GFRP delamination failure occurs, the residual sizing agent and CNTs at the fiber–resin interface, which have undulations, can act as obstacles to mechanical locking and hinder crack propagation along the interface. Therefore, the interface performance of the laminates is enhanced, and the interface debonding is suppressed. But if the CNT dispersion is excessive (above 0.7 gsm), the sizing agent on the surface of fibers is basically dissolved by acetone and detached, which is adverse to the improvement of the roughness of the fiber surface and the interfacial performance of the laminates.

The complexity of the propagation path of cracks in the interlaminar is related to the aggregation structure of CNTs. As the content of CNT increases, the aggregation structure of CNT changes from discrete particles to continuous films. In the experiment, it was found that the discontinuous CNT aggregation structure can guide cracks to deflect between layers. A more complex crack propagation path means more energy dissipation, which is beneficial for improving the interlaminar fracture toughness of GFRP. The continuous CNT layer is relatively dense, and the CNT is tightly entangled. Cracks are not easy to propagate in the toughened layer and tend to propagate towards the weaker non-toughened zone, which limits the full potential of CNT interleaves for interlaminar toughening.

In this work, CNT interleaves with a surface density of 0.5 gsm demonstrated the best toughening effect, because it has a well-bonded interface between glass fibers and resin, and CNTs aggregate into heterogenous structures between layers. The failure mode of interface debonding is suppressed. Cracks can propagate between layers and be guided and deflected by micrometer-sized CNT aggregates. Finally, the optimal interlaminar fracture toughness is obtained by combining these two aspects.

### 3.5. Comparison with the State of the Art

Table 1 summarizes the latest progress in using various interlayer toughening materials for in the literature. It should be noted that only GFRP composite materials are included here to avoid other possible impacts.

The starting point of interlaminar toughening is to improve the interlaminar fracture toughness without damaging the in-plane performance. However, the mechanical properties of interlayer toughening materials are often lower than those of fiber layers, which leads to a decrease in in-plane performance as the interlayer thickness increases. Therefore, a thinner interlayer is considered more conducive to avoiding this issue. Among all the most advanced studies listed, the performance improvement achieved by the process method we applied is leading, while the intercalation thickness we used is almost the thinnest. A 0.5 gsm CNT spray layer is semi-embedded in the glass fiber sizing agent with an original thickness of only 10–100 nm.

In addition, our method focuses on the important role of the sizing agent on the surface of commercial glass fiber woven fabrics in the production of glass fibers and the performance of composite materials. Compared with other methods of first removing the sizing and then adding the sizing, the post-treatment method of CNT spray coating is simpler in process, and the mechanical structure toughening mechanism brought by CNT introduction also has a wider range of applications.

## 4. Conclusions

In this article, heterogeneous phase toughening of bidirectional GFRP is studied. By introducing CNTs with controlled content in the interlaminar through the spray coating method, toughened layers of CNTs with different aggregation structures are obtained, along with solvent evaporation. They were used for interlaminar toughening of laminates, and the following conclusions were drawn after testing by DCB, SEM, and SPM.

The acetone solvent used for dispersing CNTs in this experiment can partially dissolve the existing sizing agent on the fiber surface. Spraying an appropriate amount of acetone is beneficial for the good bonding between CNTs, fibers, and the resin, thereby improving the bonding between fibers and resins. In addition, it was found that CNTs with a surface density of 0.5 gsm can form larger discrete particles in interlaminar region. Unlike at higher surface densities, CNTs will aggregate into continuous and dense film layers. And the discontinuous toughening layer is beneficial for guiding interlayer crack deflection and generating more energy dissipation. The results also reveal that there is an optimal amount of CNT dispersion (0.5 gsm) for toughening and modifying the laminates, which can improve the adhesion of the fiber–resin interface and suppress the debonding damage caused by crack propagation from the fiber surface. Simultaneously, discontinuous toughening layers can lead to the complexity of interlaminar crack propagation paths. In the end, the two aspects coordinated to achieve the most significant toughening effect, with an improvement of about 82% compared to the baseline.

## Figures and Tables

**Table 1:** Comparison of improvement of mode I interlaminar fracture toughness by using different interleaf materials.

Interleaf Material	Addition Amount	G_IC,ini_ Improvement (%)	G_IC,prop_ Improvement (%)	Refs.
Polycarbonate film	127 μm	-	53	[33]
Plasma-treated polycarbonate film	127 μm	-	53	[33]
CNT/epoxy film	10 μm	113	43	[34]
Toner/epoxy film	40 μm	92	23	[34]
CNT buckypaper	40 μm	39	67	[24]
CNT film	30.5 μm	−22	−73	[35]
CNT/carbon black film	33 μm	119	30	[35]
CNT/GO films	32 μm	11	2	[35]
TiO_2_ nanofiber sheets	11 μm	-	74.35	[36]
2-layer aligned CNT sheet	0.354 gsm	-	46.77	[37]
4 layer aligned CNT sheet	0.708 gsm	-	−45.16	[37]
CNT spray coating (0.3 gsm)	0.3 gsm	107.0	67.6	This work
CNT spray coating (0.5 gsm)	0.5 gsm	136.0	82.0	This work

## Data Availability

The data that support the findings of this study are available from the corresponding authors [Y.O. and D.M.] upon reasonable request.
